# A Community-Engaged Scoping Review of Affordable Housing Models for LGBTQ+ Older Adults

**DOI:** 10.1093/geront/gnaf103

**Published:** 2025-03-13

**Authors:** Angela K Perone, Leyi Zhou, Brevin Reed, Navya Singh, Sydney Kopp-Richardson, Josh Dubensky, Thomas Godwin, Jiwon Shin, Megan Lee, Ann Glusker

**Affiliations:** University of California, Berkeley, School of Social Welfare, Center for the Advanced Study of Aging Services, Berkeley, California, USA; University of California, Berkeley, School of Social Welfare, Center for the Advanced Study of Aging Services, Berkeley, California, USA; University of California, Berkeley, School of Social Welfare, Center for the Advanced Study of Aging Services, Berkeley, California, USA; University of California, Berkeley, School of Social Welfare, Center for the Advanced Study of Aging Services, Berkeley, California, USA; SAGE (Advocacy & Services for LGBTQ+ Elders), USA; SAGE (Advocacy & Services for LGBTQ+ Elders), USA; SAGE (Advocacy & Services for LGBTQ+ Elders), USA; University of California, Berkeley, School of Social Welfare, Center for the Advanced Study of Aging Services, Berkeley, California, USA; University of California, Berkeley, School of Social Welfare, Center for the Advanced Study of Aging Services, Berkeley, California, USA; University of California, Berkeley, School of Social Welfare, Center for the Advanced Study of Aging Services, Berkeley, California, USA

**Keywords:** Affordable housing, Gender, Race and ethnicity, Transgender

## Abstract

**Background and Objectives:**

LGBTQ+ older adults have heightened needs for affordable housing, given structural and systemic barriers and inequities across their lives. Little is known about affordable housing models for LGBTQ+ older adults.

**Research Design and Methods:**

This study presents the first comprehensive review of LGBTQ+ affordable housing to date. It uses an innovative multimethod community-engaged approach that combines Arksey and O’Malley’s five-step framework for scoping reviews with community-engaged data to answer the following community-driven research questions: What affordable housing models exist for LGBTQ+ older adults? How do these models address the diverse needs of LGBTQ+ older adults?

**Results:**

Data revealed ten affordable housing models for LGBTQ+ older adults: affordable housing developments, naturally occurring retirement communities, cohousing, mobile communities, homesharing, community land trusts, accessory dwelling units, limited equity cooperative housing, restored multifamily and single-family homes, and tiny homes. Affordable housing developments dominated and tended to be overrepresented by white residents. Transgender and lesbian older adults and LGBTQ+ older adults of color often led projects that invoked nontraditional housing models.

**Discussion and Implications:**

While affordable housing developments for LGBTQ+ older adults are growing, different models may be needed to address the diverse needs of this community. Intersecting experiences of trauma, discrimination, and exclusion may be driving the need and desire among transgender older adults, older lesbians, and LGBTQ+ older adults of color to develop alternative affordable housing models. Understanding the diverse needs of housing for LGBTQ+ older adults will help policymakers, practitioners, and researchers better serve this diverse community.

## Background and Objectives

LGBTQ+ older adults experience discrimination across the life course that contributes to social, health, and economic disparities (e.g., Bouton et al., 2023; Emlet, 2016; Fabbre and Gaveras, 2020; Turesky, 2022), invisibility (Perone, 2021), and also constructed communities of support (often with chosen families; e.g., Perone, 2024; Perone et al., 2025; Wardecker and Matsick, 2020) as they age. This complex interplay between challenges and community responses to challenges has created unique interventions for LGBTQ+ older adults to address disparities and discrimination. The bulk of this research focuses on health and health disparities among LGBTQ+ older adults. Very little research exists on housing for LGBTQ+ older adults, which means policymakers and practitioners have little guidance on how to best serve this population.

Existing research on housing underscores challenges for inclusive and affordable housing for LGBTQ+ older adults (Romero et al., 2020; Turesky, 2022). Single women with a median age of 78 comprise nearly 60% of households spending more than half of their income on rent (Curran, 2017; Turesky, 2022), and this worsens for lesbians, given they have fewer financial resources (Goldberg, 2009; Turesky, 2022). A 2014 report by the Equal Rights Center documented that nearly half of older same-sex couples seeking senior housing experienced discrimination, including housing refusal or higher rents. Transgender and nonbinary older adults and older adults of color experience additional challenges (Equal Rights Center, 2014). While 71% of older adults 65+ own a home, only 43% of transgender and nonbinary older adults are homeowners (AARP, 2022). Black (42%) and Latino (54%) older adults also have lower homeownership (AARP, 2022). Some LGBTQ+ older adults report interest in living in communities that comprise or target a large proportion of LGBTQ+ older adults for safety and inclusion (Romero et al., 2020). However, definitions of safety and inclusion likely vary within LGBTQ+ aging communities that could shape housing and service needs among LGBTQ+ older adults.

While scholarly research is sparse on LGBTQ+ housing for older adults, there has been significant interest in identifying solutions and housing models that work for LGBTQ+ older adults among community organizations serving this community (e.g., HOMES Coalition, 2024; LaTosch, 2023). National community organizations like SAGE have developed reports and toolkits on affordable housing for LGBTQ+ older adults. Most of this housing provides opportunities for aging-in-place and aging-in-community that connect LGBTQ+ older adults to culturally responsive and inclusive physical spaces and services. Researchers and community organizations are also exploring alternative models of inclusive housing for LGBTQ+ older adults to age-in-place and age-in-community such as cohousing, community land trusts (CLTs), tiny homes, and mobile communities (e.g., Baldwin et al., 2019). However, the scholarly research is thin and has not been systematically examined to provide guidance for practitioners or policymakers interested in this area.

This article has several goals. First, it addresses gaps in knowledge about affordable housing models for LGBTQ+ older adults by asking two questions (1) What affordable housing models exist for LGBTQ+ older adults? (2) How do these models address the diverse needs of LGBTQ+ older adults? Second, it presents an innovative model for community-engaged scoping reviews that incorporates both scholarly and community-based expertise. These research questions arose from needs identified by community partners, and the research design reflects a collaborative, community-engaged approach to addressing scholarly gaps that can be supplemented by community-based knowledge. Third, this article contributes to ongoing conversations about potential interventions and future directions and concludes with research, practice, and policy recommendations.

## Research Design and Methods

This study integrates traditional scoping review methodologies with community-engaged research approaches. It enhances the scholarly scoping review by incorporating a gray literature review and community data from two reports provided by the only national community organization focused on housing for LGBTQ+ older adults. This national partner is the largest and oldest nonprofit focused on LGBTQ+ aging and the only national nonprofit foregrounding housing for LGBTQ+ older adults. We applied an innovative method that combines the scoping review framework developed by Arksey and O’Malley (2005) and further refined by Levac et al. (2010) that is supplemented by community-engaged research approaches that elevate community-based knowledge and expertise. Here, the community-engaged research incorporated monthly (and sometimes biweekly) meetings between the research team and community partner to refine the research questions, research design, data collection and analysis, and dissemination. Community data includes information from these community meetings about hard-to-access and sometimes not publicly available gray literature and community data from two community partner reports about emerging affordable housing models for LGBTQ+ older adults. This approach ensures the rigor and comprehensiveness of our literature search and selection processes while also elevating community-based knowledge. One innovation of this study is the blending of these research approaches. Traditional methodological approaches would separate a scoping review from community-based data and often from other gray literature. But we believed strongly that the community-driven research questions would be best answered by blending data from scholarly databases, gray literature, and community-based data, which also allows us to contextualize and combine relevant data and keep it in conversation with one another. A scoping review was selected over another review (e.g., systematic, narrative) because the research questions were exploratory and involved rigorous and systematic knowledge synthesis to map key concepts, evidence, and gaps in research (Heyn et al., 2019).

### Search Strategy

Given limited scholarship on LGBTQ+ housing, the search included peer-reviewed research in English from January 1997 to March 2024 and encompassed any study on affordable housing models for LGBTQ+ older adults. To ensure the accuracy and reliability of our results, we conducted a comprehensive electronic search across seven databases: ProQuest Social Science (PsycINFO, Sociological Abstracts, and Social Services Abstracts), PubMed, Scopus, and LGBT + Life. Search strategies captured programs, concepts, and models related to housing for LGBTQ+ older adults. The team received additional guidance from an academic librarian with expertise in systematic reviews. Keywords were divided into three categories: LGBTQ+ (LGBT, Lesbian, Gay, Bisexual, Transgender, Queer, etc.); Older Adults (Older adult, Older, Adult, Aging, Elderly/Elder, Senior Citizen/Senior); and Community-Based Housing (Community-Based Housing, Co-housing, Homeshare, Independent Living, etc.). The initial search process was carried out by LZ, who subsequently downloaded a total of 1,340 articles. After deleting duplicates, 1,014 peer-reviewed articles remained.

### Eligibility Criteria

The inclusion and exclusion criteria and data extraction format were drafted by LZ and AP, reviewed, and finalized in coordination with the coauthors, with guidance from an academic librarian specializing in scoping and systematic reviews. See Supplementary Material for search strings used in the electronic databases.

Articles were included based on the following criteria: (a) Study Design: Any study design (qualitative, quantitative, case study). Nonempirical articles like reports, perspectives, or op-eds were moved to the gray literature review, when relevant, but excluded from the scholarly scoping review. (b) Population: The target population included LGBTQ+ older adults (40+). While 40 is generally considered young for “aging” research, we incorporated scholarship about housing for LGBTQ+ older adults as young as 40, given research on accelerated aging for multiply minoritized populations (e.g., Pereira and Banerjee, 2021; Valencia et al., 2023). Our community partner also provided expertise about the experiences of transgender aging, especially low-income and BIPOC transgender adults, that suggested lowering the age to 40. (c) Topic: The main topic of the literature needed to focus on community-based housing, which includes all community housing models except institutional living (i.e., nursing homes). Assisted living was included because it is often considered community-based housing through Medicaid waiver programs. Other potential models include coshared housing, homesharing, tiny houses, CLTs, mobile homes, retirement communities, affordable housing developments, and so forth. This list was created with a national community partner and academic librarian.

Articles were excluded if: (a) they were review articles (scoping review, systematic review, meta-analysis, etc.) or study protocols; (b) focused on housing for youth or younger adults; (c) did not target LGBTQ+ communities in the United States; (d) focused only on institutional housing; or (e) only addressed luxury or market-rate housing.

### Identifying the Scholarly and Nonscholarly Literature

**Figure 1.**
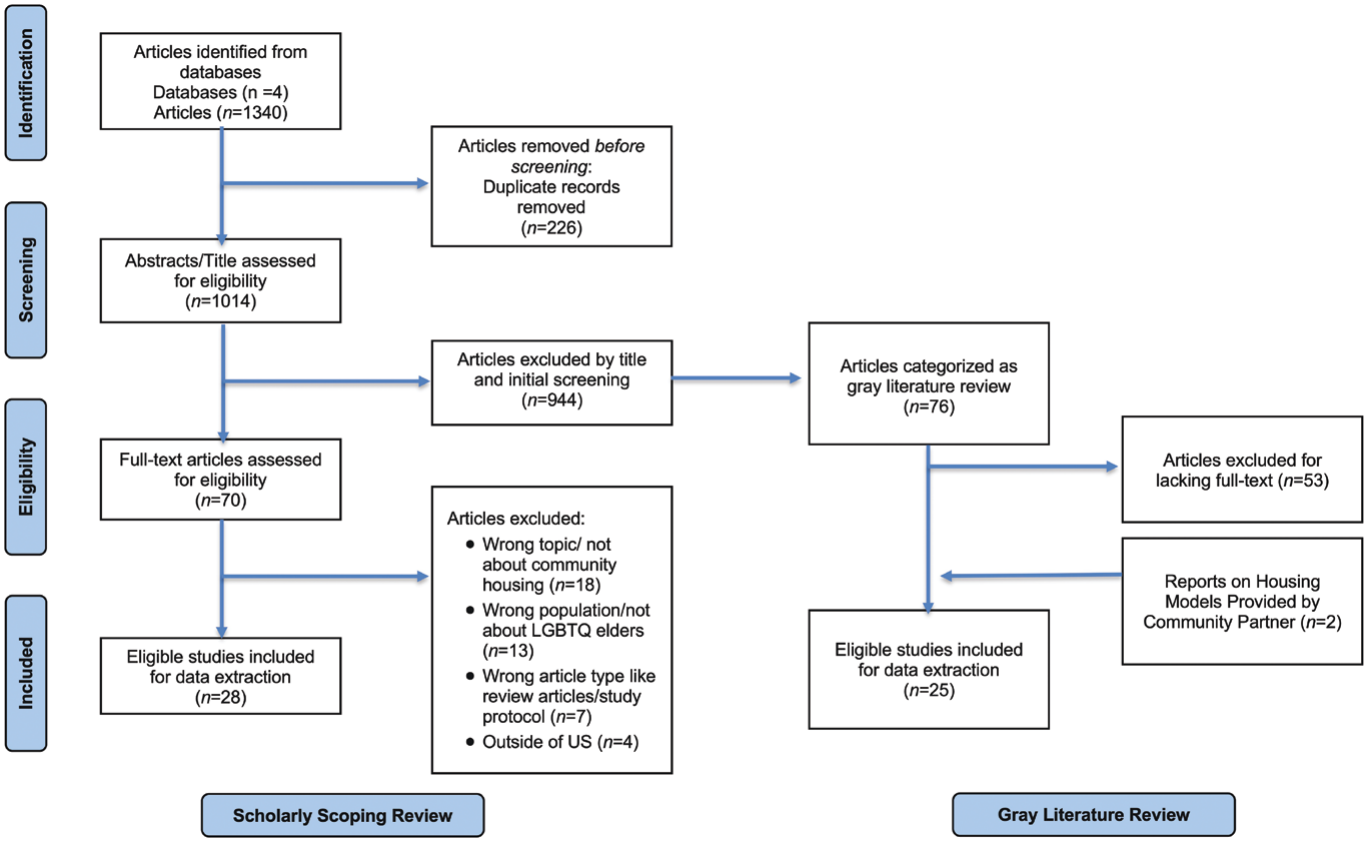
PRISMA flow diagram.

The study screening was conducted on the platform Rayyan. Using the above-mentioned search strategy, 1,340 articles were identified as potentially relevant: ProQuest Social Sciences (PsycINFO, Sociological Abstracts, and Social Services Abstracts, 443 articles); PubMed (180 articles); Scopus (559 articles); and LGBT + Life (158 articles). After removing 226 duplicates, the remaining 1,014 articles were screened by reading the title and abstract. The review followed two steps for study selection. First, two independent reviewers (BR and NS) screened the titles and abstracts of retrieved studies for eligibility. A third reviewer (AP) resolved conflicts, which left 70 papers that met the inclusion and exclusion criteria. Among the 944 articles excluded, 76 were categorized as gray literature and moved to a gray literature review. Second, two reviewers (BR and NS) independently read and screened full texts of scholarly articles from the first step. A third reviewer (AP) resolved conflicts, resulting in 28 papers included and 42 papers excluded. Eighteen articles were excluded because their topic was not about community housing; 13 articles did not focus on LGBTQ+ older adults; 7 articles were review articles or study protocols; and 4 articles focused on populations outside the US. A fourth reviewer (LZ) reviewed the 76 potential gray literature abstracts and obtained the full text of 23 articles, which were selected for the next round of data extraction. The final dataset included 28 scholarly articles and 23 nonscholarly articles (gray literature), plus two reports on LGBTQ+ housing models from our community partner for data extraction and analysis. Figure 1 (PRISMA Flow Diagram) shows the process for searching and selecting literature for the scholarly scoping review and gray literature review.

### Data Extraction, Analysis, and Synthesis

Two reviewers (BR and NS) conducted data extraction for 28 scholarly articles. Another two reviewers (ML and JS) conducted data extraction for 25 nonscholarly articles. Both sets of data were reviewed and finalized by a third reviewer (AP). We created two Excel spreadsheets: one for data extraction from scholarly literature and another for nonscholarly literature. The data extraction items in both spreadsheets were selected by the first author (AP) and the second author (LZ) based on the study protocol and were discussed with the entire team.

Given the two forms of data (i.e., scholarly and gray literature), we separated the findings into two tables. The spreadsheet for scholarly articles includes housing models, study method, sample, target age, and whether the scholarly article discusses implications for transgender communities, BIPOC communities, and unhoused communities. See Table 1: Scholarly Scoping Review Housing Models and Populations.

**Table 1.** Scholarly Scoping Review for Housing Models and Populations

Authors	Housing type	Sample (*n*)	Study method	Target age group	Transgender	BIPOC	LGBTQ+ homelessness
Adelman et al., 2006	Affordable housing development	*n* = 1301 (LGBT adults ages 18–92)	Mixed methods	18+	N/A	N/A	N/A
Boggs et al., 2017	NORC	*n* = 73 (lesbian or gay ages 50–69, and lived with a partner)	Qualitative	Older LGBTQ adults; no detailed information on target age group	N/A	N/A	N/A
Bradford et al., 2016	Cohousing	*n* = 26 (lesbians with mean age of 68, age range of 64–71)	Mixed methods	Older lesbians; no detailed information on target age group.	N/A	N/A	N/A
Dixon, 2018	Affordable housing development; NORC; mobile community	Not reported	Case study; descriptive	Not reported	N/A	N/A	N/A
Glick et al., 2019	Affordable housing development	*n* = 17 (ages 23 to 39 with one 70-year-old who was transgender or nonbinary)	Qualitative	Transgender; no target age group	N/A	N/A	Yes
Hamburger, 1997	Affordable housing development; cohousing; homesharing	*n* = 44 (nonheterosexual older adults in Bay Area; median age of 59.5)	Mixed methods	Nonheterosexual; no detailed information on target age group	N/A	N/A	N/A
Johnston and Meyer, 2017	Affordable housing development	Not reported	Quantitative	Older LGBTQ adults age 60+	N/A	N/A	N/A
Kilbourn, 2016	Affordable housing development	Not reported	Case study; descriptive	Older LGBTQ adults; no detailed information on target age group	N/A	N/A	N/A
Larson, 2016	Affordable housing development	Not reported	Case study; descriptive	Older LGBT older adults; no detailed information on target age group	N/A	N/A	N/A
Matthews et al., 2015	Affordable housing development; mobile community	Not reported	Case study; descriptive	Older LGBTQ adults; no detailed information on target age group	N/A	N/A	N/A
Matthews et al., 2017	Affordable housing development	Not reported	Case study; descriptive	Older LGBTQ adults; no detailed information on target age group	N/A	N/A	N/A
Merrick, 2010	Affordable housing development; NORC	*n* = 34 (retirement-age lesbian and gay men from three different Florida retirement communities)	Qualitative	LGBT older adults at retirement-age; no detailed information on target age group	N/A	N/A	N/A
Orel, 2006	Affordable housing development	*n* = 26 (LGB elders age 65+)	Qualitative	Older gay and lesbian 65+	N/A	N/A	N/A
Putney et al., 2018	Affordable housing development	*n* = 50 (sexual and gender minority (SGM) older adults ages 55 and over)	Qualitative	Older LGBTQ adults; no detailed information on target age group	Yes	N/A	N/A
Putney et al., 2020	Affordable housing development	*n* = 50 (sexual and gender minority (SGM) older adults ages 55 and over)	Qualitative	Older LGBTQ adults; no detailed information on target age group	N/A	N/A	Yes
Quigley, 2017	Affordable housing development	Not reported	Case study; descriptive	Older LGBTQ adults; no detailed information on target age group	N/A	N/A	N/A
Ranahan, 2017	Affordable housing development; homesharing	*n* = 7 (program directors and/or development managers from residential services for LGBT older adults)	Qualitative	Older LGBTQ adults; no detailed information on target age group	N/A	N/A	N/A
Rivera et al., 2011	Affordable housing development	*n* = 15 (older gay and lesbian, ages 61–92)	Qualitative	Older LGBTQ adults; no detailed information on target age group	N/A	N/A	N/A
Rosenwohl-Mack et al., 2022	Affordable housing development	*n* = 21 (LGBTQ + welcoming senior housing)	Qualitative	Older LGBTQ adults; no detailed information on target age group	Yes	Yes	Yes
Savage and Barringer, 2021	Affordable housing development	*n* = 1,762 (LGBT Americans ages 45 and older)	Mixed methods	Older LGBTQ adults; no detailed information on target age group	N/A	N/A	N/A
Steele, 2022	Affordable housing development	*n* = 6 (LGBTQ+ adults ages 65 + from the Louisville metro area in Kentucky)	Mixed methods	Older LGBTQ adults age 65+	N/A	N/A	Yes
Sullivan, 2011	Affordable housing development	*n* = 22 (current residents in LGBTQ housing for older adults)	Mixed methods	Older LGBTQ of retirement age (50s to 80s)	N/A	N/A	N/A
Sullivan, 2014	Affordable housing development	*n* = 38 (current residents in LGBTQ housing for older adults)	Qualitative	Older LGBTQ of retirement age (50s to 80s)	N/A	N/A	N/A
Turesky, 2022	Affordable housing development	Not reported	Descriptive	Older LGBTQ adults; no detailed information on target age group	N/A	N/A	Yes
Urness, 2016	Affordable housing development; cohousing; mobile community	*n* = 15 (nonheterosexual adults over age of 50)	Mixed methods	Older LGBTQ adults; No detailed information on target age group	N/A	N/A	N/A
White and Gendron, 2016	Affordable housing development	Not reported	Case study	Older LGBTQ adults; no detailed information on target age group	Yes	N/A	N/A
White and Martin, 2010	Mobile community	Not reported	Qualitative	Retired/semiretired women who were mostly queer; no detailed information on target age group	N/A	Yes	N/A
Woody, 2016	Cohousing	Not reported	Case study; descriptive	LGBTQ/SGL older adults 60+	N/A	N/A	N/A

*Notes*: BIPOC = XXX; LGBTQ = xxx; LGBTQ+ = XXX; *N* = sample size; N/A = XXX; NORC = xxx.

The spreadsheet for nonscholarly articles (gray literature) includes housing models, the city and state of the housing model, and whether the article discusses implications for transgender communities, BIPOC communities, and unhoused communities. See Table 2: Gray Literature Review Housing Models and Populations.

**Table 2.** Gray Literature Review for Housing Models and Populations

Title	Source	Housing type	City/state	Transgender	BIPOC	LGBTQ+ homelessness
Aging gay population fuels retirement community market (2006)	NBC News	Affordable housing development	General	N/A	N/A	Yes
Bajko, M. S. (2012)	Bay Area Reporter	Affordable housing development	San Francisco, CA	N/A	N/A	Yes
Bajko, M. S. (2021)	Bay Area Reporter	Affordable housing development	San Francisco, CA	N/A	N/A	Yes
Chaitman, S. (2011)	Windy City Times	Affordable housing development	Chicago, IL	N/A	N/A	Yes
COMMUNITY / Director helps plan home for gay seniors. (2002)	SFGate	Affordable housing development	San Francisco, CA	N/A	N/A	Yes
Ferrannini, J. (2021)	Bay Area Reporter	Affordable housing development	Bay Area, CA Brooklyn, NY	N/A	Yes	Yes
Forman, R. (2015)	Windy City Times	Affordable housing development	Chicago, IL	N/A	N/A	Yes
Hammond, G. R. (2016)	Windy City Times	Affordable housing development	Chicago, IL	N/A	N/A	N/A
Kimura, D. (2014)	Windy City Times	Affordable housing development	Chicago, IL	N/A	N/A	Yes
LaTosch, K. (2023)	Between The Lines	Affordable Housing Development	Philadelphia, PA Los Angeles, CA MI	N/A	N/A	Yes
Lavers, M. K. (2011)	Windy City Times	Affordable housing development; homeshare	Chicago, IL Hollywood, CA Philadelphia, PA San Francisco, CA	N/A	N/A	Yes
LGBT-friendly senior housing land transfer OK’d. (2013)	Windy City Times	Affordable housing development	Chicago, IL	N/A	N/A	N/A
Michael, J. A. (2023a, 2023b)	Between The Lines	Affordable housing development	MI	N/A	N/A	Yes
Proxmire, C. A. (2011a)	Between The Lines	Affordable housing development; homesharing	Los Angeles, CA Chicago, IL	N/A	N/A	Yes
Proxmire, C. A. (2011b)	Between The Lines	Affordable housing development	Ferndale, MI	N/A	N/A	Yes
Report: Unique Challenges Face Older Gay Residents. (2015)	Between The Lines	Affordable Housing Development	MA	Yes	N/A	Yes
Saunders, D. (2023)	Between The Lines	Affordable housing development	Ferndale, MI	N/A	N/A	Yes
Scratching the surface (2014)	Philadelphia Gay News	Affordable housing development; naturally occurring retirement community	Philadelphia, PA	N/A	N/A	Yes
Simonette, M. (2018)	Windy City Times	Affordable housing development	Chicago, IL	N/A	N/A	Yes
Tunney, Heartland (2012)	Windy City Times	Affordable housing development	Chicago, IL	N/A	N/A	Yes
Wasserman, M. (2015)	Windy City Times	Affordable housing development	Chicago, IL New York City, NY	N/A	N/A	Yes
Where will you live? (2012)	Philadelphia Gay News	Affordable housing development; naturally occurring retirement community	Philadelphia, PA San Francisco, CA Los Angeles, CA	N/A	N/A	Yes
Woyton, M. (2023)	Windy City Times	Affordable housing development	White Plains, NY	N/A	N/A	Yes
**Gray Literature: Reports from Community Partner**						
SAGE (2020)	SAGE/National LGBTQ + Elder Housing Initiative	Affordable housing development	General Chicago, IL Los Angeles, CA Minneapolis, MN New York City, NY Philadelphia, PA Sacramento, CA San Francisco, CA San Diego, CA	Yes	Yes	Yes
SAGE (n.d.)	SAGE/National LGBTQ+ Elder Housing Initiative	Cohousing; community land trust; limited equity cooperative housing; homesharing; accessory dwelling units; tiny homes	General Durham, NC New York City, NY Memphis, TN	Yes	Yes	Yes

*Notes*: BIPOC = XXX; LGBTQ = xxx; LGBTQ+ = XXX; N/A = XXX.

The authors compared data extraction results from the 53 sources, summarizing and analyzing the literature. The team included subteams that each met regularly (weekly) and then met collectively to discuss and compare data analysis and findings. The team also produced research memos to help synthesize the data.

## Results

This community-engaged scoping review has several important findings about affordable housing models for LGBTQ+ older adults—and potential new directions for research on homeless prevention for LGBTQ+ older adults.

### What Affordable Housing Models Exist for LGBTQ+ Older Adults?

Data revealed 10 housing models that were being used to address affordable housing needs for LGBTQ+ older adults, including those experiencing housing precarity: affordable housing developments, naturally occurring retirement communities (NORCs), cohousing, mobile communities, homesharing, CLTs, accessory dwelling units (ADUs), limited equity cooperatives, restored multifamily and single-family homes, and tiny homes. The bulk of the scholarly (and gray) literature focused on affordable housing developments, but some literature also discussed NORCs, cohousing, mobile communities, homesharing, and CLTs. We identified four additional housing models from gray literature and community data provided by our community partner, SAGE: ADUs, limited equity cooperatives, restored multifamily and single-family homes, and tiny homes.

#### Affordable housing developments

Affordable housing developments for LGBTQ+ older adults dominated the literature. Of the 53 articles included in this review, 47 of the articles (over 88%) discussed LGBTQ+ affordable housing developments. Six articles discussed the first LGBTQ+ affordable housing development—Triangle Squares Apartment—that opened in Los Angeles in 2007 (Dixon, 2018; Johnston and Meyer, 2017; Mathews et al., 2015; Proxmire, 2011a; SAGE, 2020; Turesky, 2022). In 2022, 70% of the residents were LGBTQ+ and every resident was at least 62 years old (SAGE, 2020). One-third of the units are set aside for people living with HIV, and about one-third have Project-Based Section 8 subsidies (SAGE, 2020).

While Triangle Squares opened first, more articles (*n* = 14) discussed an LGBTQ+ affordable housing development in Chicago: Town Hall Apartments (Chaitman, 2011; Dixon, 2018; Forman, 2015; Hammond, 2016; Johnston and Meyer, 2017; Kimura, 2014; Lavers, 2011; LGBT-friendly senior, 2013; Mathews et al., 2017; Proxmire, 2011a; SAGE, 2020; Simonette, 2018; Tunney, Heartland, 2012; Wasserman, 2015). Town Hall Apartments opened in 2014 and provides 100% affordable housing for adults 55 and over (SAGE, 2020). As of 2020, 60% of its residents identified as LGBTQ+ (SAGE, 2020).

Ten articles discussed Openhouse’s affordable housing developments at 55 Laguna and 95 Laguna in San Francisco (Adelman et al., 2006; Bajko, 2012, 2014, 2020; Dixon, 2018; Johnston and Meyer, 2017; Kilbourn, 2016; SAGE, 2020; SF Gate, 2002; Turesky, 2022). 55 Laguna was completed in March 2017, and 95 Laguna was completed in July 2019 and provide residential units for adults 55+ (55 Laguna) or 62+ (95 Laguna) and who have a household income that does not exceed 50% of the Area Median Income (SAGE, 2020). Openhouse’s affordable housing also has 14 of its 117 units set aside for people who were formerly unhoused, and 15 units for unhoused people placed by San Francisco’s Department of Homelessness Continuum of Care (COC) program (SAGE, 2020).

Four articles discussed LGBTQ+ housing developments in New York City (Dixon, 2018; SAGE, 2020; Wasserman, 2015; Woyton, 2023). Stonewall House in Brooklyn opened in 2019. Crotona Pride House in the Bronx opened in 2020. Both are 100% affordable housing developments for residents 62+ with 50% and below Area Median Income and Project Based Section 8 Housing Choice Vouchers (SAGE, 2020). Thirty percent of Crotona’s units and 25% of Stonewall’s units are set aside for people who were formerly unhoused (SAGE, 2020).

Four articles discussed the need and process for LGBTQ+ affordable housing developments in Michigan (LaTosch, 2023; Michael, 2023a; Proxmire, 2011b; Saunders, 2023). None of these were scholarly articles, which may be, in part, because LGBTQ+ affordable housing has not opened yet. The Raymond E. Shepherd House in Ferndale, Michigan (near Detroit) broke ground in 2023 and will only include affordable housing units (Michael, 2023b).

Six articles described LGBTQ+ affordable housing developments elsewhere in the United States, including Spirit on Lake in Minneapolis (Dixon, 2018; Johnston and Meyer, 2017; SAGE, 2020), John C. Anderson Apartments in Philadelphia (Dixon, 2018; Johnston and Meyer, 2017; Lavers, 2011; SAGE, 2020; Scratching the surface, 2014; Where will you live, 2012), North Park Senior Apartments in San Diego (Johnston and Meyer, 2017; SAGE, 2020), and Lavender Courtyard in Sacramento (Johnston and Meyer, 2017; SAGE, 2020). Spirit on Lake Apartments opened in 2014, and 65% of its residents are LGBTQ+ people; units are restricted to those at 50% and below Area Median Income (SAGE, 2020). John C. Anderson Apartments opened in 2014 and serves people 62+ with incomes 60% or below Area Median Income; several units are reserved for people with extremely low or very low income (SAGE, 2020). North Park Senior Apartments opened in 2017, and 100% of its units are affordable housing serving older adults 55+ (SAGE, 2020). Ten percent are also designated for people formerly unhoused (SAGE, 2020). Lavender Courtyard provides affordable housing for very and extremely low-income older adults 62+ (SAGE, 2020). Twenty-four of its 53 units are reserved for Project-Based Vouchers for households exiting homelessness (SAGE, 2020).

While affordable housing developments represented the bulk of the literature, many articles also noted limitations of this model for serving LGBTQ+ older adults experiencing housing precarity (e.g., Johnston and Meyer, 2017; Putney et al., 2020; Turesky, 2022; Urnes, 2016). First, the housing need far outweighs the availability. Second, because of antidiscrimination housing laws, they cannot expressly include only LGBTQ+ older adults (Johnston and Meyer, 2017). SAGE has developed toolkits, trainings, and guidance that can help communities increase the proportion of its LGBTQ+ residents, while maintaining access and support for all individuals, regardless of sexual orientation and/or gender identity. While residents’ sexual orientation and gender identity is not always reported, a 2020 report noted that 40% of the residents in Chicago’s Town Hall Apartments, 30% of the residents in Los Angeles’ Triangle Square Apartments, and 35% of the residents in Minneapolis’s Spirit on Lake Apartments do not identify as LGBTQ+ (SAGE, 2020).

#### Naturally occurring retirement communities

NORCs represent housing communities that were not planned for older adults but over time become places that disproportionately house older adults (Ahrentzen and Steiner, 2019). NORCs may be housing-based (e.g., located in a single apartment building, housing complex), neighborhood-based (e.g., single-family homes), or community-based that represent a larger area beyond a neighborhood (Ahrentzen and Steiner, 2019). Some NORCs also provide community programs, social services, transportation, housekeeping, and group activities (Ahrentzen and Steiner, 2019). NORCs are more likely to include individuals who are older, less economically secure, and have more functional limitations than other housing developments or Village models (e.g., Ahrentzen and Steiner, 2019; Greenfield, 2012).

Two articles described NORCs (Boggs et al., 2017; Dixon, 2018). Some LGBTQ+ older adults may have incomes too high to qualify for affordable housing (Dixon, 2018) but may be in acute crisis (e.g., death of a partner and loss of their income; health issue) from losing their housing. NORCs can reduce costs by leveraging community-based networks, services, and programs that can help LGBTQ+ older adults remain at home (Boggs et al., 2017).

#### Cohousing

Cohousing represents an intentional housing community that often includes several private homes built around a common green space and common buildings shared by residents (SAGE, n.d.). Cohousing provides social connection, community support, shared governance, and opportunities to age-in-place. One article included data from LGBTQ+ older adults expressing a desire for shared spaces like those in cohousing to build community (Urnes, 2016), and another article described cohousing more generally among LGBTQ+ older adults (SAGE, n.d.). However, cohousing models often require significant upfront financial investment and time commitment (SAGE, n.d.), and one article noted that a women’s cohousing group had been working for several years to acquire property for intergenerational cohousing for women (Hamburger, 1997). Another article discussed plans to incorporate principles of cohousing for LGBTQ+/same-gender-loving older adults of color in Washington, DC (Woody, 2016). Once funding has been secured, cohousing may provide stable housing for LGBTQ+ older adults who may lack other housing options and might otherwise experience housing precarity.

#### Mobile communities

Five articles discussed LGBTQ+ communities with mobile homes or recreational vehicles (RV; Dixon, 2018; Mathews et al., 2015; Merrick, 2010; Urness, 2016; White and Martin, 2010). Every community was designed for and/or by older lesbians. Bayside Park in Florida was created by a lesbian couple in 1997 as a “women’s community” with manufactured homes and RVs (Merrick, 2010). Ages ranged from 43 to 75 and averaged 61 (Merrick, 2010). The Resort on Carefree Boulevard in Fort Myers, Florida includes mobile homes for lesbians (Dixon, 2018; Mathews et al., 2015). Discovery Bay in Washington hosts lesbian-friendly RVs (Dixon, 2018). In an interview with an older Black lesbian RV resident, Vera describes how it provides a sense of community support when someone gets ill but also notes that she was one of only a few women of color there (White and Martin, 2010).

#### Homesharing

Supported homeshare matches are formal shared living arrangements between two (or more) unrelated people who live together and mutually benefit from the exchange of rent and/or services and nonprofit support. A home provider (often an older adult) shares space in their home (e.g., a spare room) or on their property with a home seeker in exchange for money, services/support, or a combination of both. A nonprofit homeshare program facilitates the match to create a safe and sustainable living situation for the parties. Four articles discussed homesharing among LGBTQ+ older adults (Hamburger, 1997; Lavers, 2011; Ranahan, 2017; SAGE, n.d.). Ranahan (2017) notes the existence of an “innovative” homesharing program that uses a screening process, background check, and ongoing support (p. 165; all core features of supported homesharing). Hamburger (1997) discusses the evolution of a Bay Area homeshare program. The third article describes homesharing as a “win-win” situation for housing affordability and caregiving (Lavers, 2011). Homesharing may support LGBTQ+ older adults who are precariously housed by providing safe, supported, and vetted matches that can also minimize risks for bias and discrimination. Homesharing can also keep LGBTQ+ older adults in their homes, including home providers who may not have other places to live or finances to pay for new housing, or who may need additional caregiving support that can be provided by a home seeker in exchange for lower rent.

#### Community land trusts

A CLT creates affordability in communities experiencing displacement and gentrification (SAGE, n.d.). It developed, in part, to provide stable housing and farmland for Black sharecroppers who lost their homes and jobs after voting (Zipman, 2024). A CLT is usually a nonprofit organization that buys land and property to create an affordable housing community by selling homes or residential units at affordable prices (SAGE, n.d.). The CLT, however, retains ownership of the land. One article referenced a project in Oakland to provide single-family homes as property held in public trust to reclaim local neighborhoods that had experienced gentrification (Ferrannini, 2021). This project provides affordable housing for LGBTQ+/same-gender-loving older adults of color who experienced an influx of white residents and subsequent spikes in housing price costs. In New York, Interboro Community Land Trust was formed in 2016 to provide lower-income housing (SAGE, n.d.).

#### Accessory dwelling units

None of the literature from the databases discussed ADUs, but ADUs were identified as an affordable housing model for LGBTQ+ older adults in SAGE’s community data reports. ADUs are smaller living units usually built on property that already has a larger home or building (SAGE, n.d.). ADUs can provide affordable housing to live near chosen family. Zoning laws in many cities can create barriers in creating ADUs, but many communities relaxed these regulations during the COVID-19 pandemic and have continued to loosen regulations to address a housing crisis in many parts of the United States. For example, the Durham Community Land Trust began building ADUs to provide more affordable housing amidst rising housing prices in Durham, North Carolina (SAGE, n.d.).

#### Limited equity cooperative housing

None of the literature from the databases discussed Limited equity cooperative housings (LECs), but this model was identified as an affordable housing model for LGBTQ+ older adults in SAGE’s community data reports. LECs provide deep affordability to those with significant housing barriers (SAGE, n.d.). Community members usually create a nonprofit cooperative that owns and controls housing and related facilities in a building and leases homes or units to residents. Unlike a CLT, residents own a portion of the co-op, instead of a home. Residents have voting and decision-making authority about what happens with the building, and LECs usually have resale restrictions to maintain affordable housing (SAGE, n.d.). LECs can help LGBTQ+ older adults who may have low credit scores, whose income is too low to afford other housing, or who may otherwise experience housing discrimination in other affordable housing spaces.

#### Restored multifamily or single-family homes

None of the literature from the databases discussed restored multifamily or single-family homes, but restored homes were identified as an affordable housing model for LGBTQ+ older adults in SAGE’s community data reports. This housing model involves community members who collectively rehab or renovate an inexpensive house. That house may be donated by a community member or purchased by community or nonprofit fundraising. House of Tulip in the New Orleans area is raising funds to restore a multifamily and multigenerational property for transgender adults (SAGE, n.d.). Zami Nobla, a national organization of Black lesbians on aging, was gifted the childhood home of a community member and Board Member that needed significant renovations. In exchange for doing the renovations (some of which comprised donated time or discounted materials), the organization was able to provide affordable housing to an older Black lesbian couple in Atlanta (Duncan, 2019; SAGE, n.d.).

#### Tiny homes

None of the literature from the databases discussed tiny homes, but tiny homes were identified as an affordable housing model for LGBTQ+ older adults in SAGE’s community data reports. My Sistah’s House—an organization run by Black trans women—built seven tiny homes for Black trans women who were facing significant challenges securing safe, affordable, and accessible housing for Black trans women, including older adults (SAGE, n.d.). My Sistah’s House operates similar to a CLT and owns the land with the tiny homes. Residences can lease or purchase tiny homes at affordable rates. It also provides transitional housing for transgender adults who are unhoused, so they can live with other transgender adults safely (SAGE, n.d.).

### How Do These Models Address the Diverse Needs of LGBTQ+ Older Adults?

Demographic data was sparse in the scholarly and gray literature. However, the literature provided important indicators of how various models may better serve various segments of the LGBTQ+ aging population, particularly relating to income and financial resources, race and ethnicity, gender, gender identity, disabilities or health conditions, and the intersections of these positionalities.

One significant challenge for LGBTQ+ older adults remains affordability. Even in LGBTQ+ housing developments with affordable housing, affordability is becoming harder to attain, and some developments are seeing shrinking proportions of low-income LGBTQ+ communities. For example, about 70% of Triangle Squares residents lived at or below the poverty level closer to when it first opened (Dixon, 2018). Years later, only 20% of its units represented 60% or below the Area Median Income (SAGE, 2020). While these numbers do not compare the same data, they reflect a shifting economic trend that affordable housing developments, especially those with mixed-income housing, provide fewer opportunities for low-income LGBTQ+ older adults who need affordable housing or are precariously housed.

Several articles also discussed concerns that LGBTQ+ affordable housing developments are located in cities or neighborhoods that are predominantly white and may not serve as many LGBTQ+ older adults of color (e.g., Ferrannini, 2021; Turesky, 2022) or address the specific needs of women (e.g., White and Martin, 2010) or transgender older adults (e.g., Putney et al, 2018). Two scholarly articles (Rosenwohl-Mack et al., 2022; White and Martin, 2010) and two gray literature articles (Ferrannini, 2021; SAGE, 2020) included older adults of color into their analysis and/or discussion about affordable housing models for LGBTQ+/same-gender loving older adults. Three scholarly articles (Putney et al., 2018; Rosenwohl-Mack et al., 2022; White and Gendron, 2016) and two gray literature articles (Report: Unique Challenges Face Older Gay Residents, 2015; SAGE, 2020) incorporated transgender older adults into their analysis and/or discussion about affordable housing models for LGBTQ+ older adults.

Articles discussing mobile communities revealed that many of these housing models are led by and often created for older lesbians (Dixon, 2018; Mattews et al., 2015; Urness, 2016; White and Martin, 2010); although, they may also lack much racial diversity (e.g., White and Martin, 2010). Several tiny homes, cohousing communities, and restored homes were led by LGBTQ+/same-gender-loving older adults of color (e.g., SAGE, n.d.; Woody, 2016). CLTs present opportunities to combat gentrification and displacement with safe and affordable housing for LGBTQ+ older adults, especially communities of color (SAGE, n.d.). Homesharing presents a model that was lauded for serving LGBTQ+ older adults with disabilities or caregiving needs (e.g., Hamburger, 1997; Lavers, 2011; Ranahan, 2017). ADUs and Limited Equity Cooperatives present additional opportunities that could serve the diverse needs of LGBTQ+ older adults (SAGE, n.d.); although, the literature has not yet addressed the gaps that these models could fill in addressing the need for affordable housing for particular communities within a larger LGBTQ+ aging population.

## Discussion and Implications

The results from this scoping review present the first comprehensive and systematic review of LGBTQ+ affordable housing to date. As such, it provides new and timely information that researchers, practitioners, and policymakers can use to better understand this area for programming and policy. Prior presentations of this paper generated significant interest, especially among practitioners, LGBTQ+ community members, and researchers studying LGBTQ+ aging who are eager for more guidance in this area, particularly as the needs for affordable housing for LGBTQ+ older adults continue to grow.

LGBTQ+ affordable housing developments have received the most attention for addressing housing needs of low-income LGBTQ+ older adults. They serve an important need in providing housing, and many also provide much-needed services and social support for LGBTQ+ older adults. However, they address a small fraction of the need. Only a handful of developments set aside specific units for people who are unhoused (and thus in dire need of affordable housing), and some have seen a decrease in affordable units overall. Laws also restrict options for designating these spaces as exclusively LGBTQ+ (Johnston and Meyer, 2017; Turesky, 2022), and many include a sizable portion of non-LGBTQ+ residents. Some LGBTQ+ communities have also expressed concerns that these developments are located primarily in white neighborhoods or tend to serve more white residents and may not always meet the needs of a racially and ethnically diverse LGBTQ+ community (e.g., Ferrannini, 2021; Turesky, 2022). Transgender older adults, older lesbians, and LGBTQ+ older adults with disabilities have expressed similar concerns about affordability, access, and inclusion at some LGBTQ+ housing developments (e.g., Mattews et al., 2015; Putney et al., 2018; Rosenwohl-Mack et al., 2022; Turesky, 2022; Urness, 2016; White and Gendron, 2016; White and Martin, 2010). Additional housing models, thus, have emerged to address the diverse needs of an aging LGBTQ+ population, including cohousing, mobile communities, homesharing, CLTs, ADUs, limited equity cooperatives, restored homes, and tiny homes.

While literature remains sparse on nontraditional affordable housing models (outside affordable housing developments), community partner data suggests that many of these housing communities are led by and/or developed for LGBTQ+ older adults who are often excluded from traditional forms of affordable housing (SAGE, n.d.). It is possible that some communities are proactively creating their own affordable housing models that provide a stronger sense of community, safety, or lived experiences based on race, gender, gender identity, or other positionalities (and their intersections). For example, tiny homes and restored homes by and for Black trans women may reflect both a desire to build community of support for and by Black trans women (e.g., My Sistah’s House in Memphis; House of Tulip in New Orleans; SAGE, n.d.) that also reflects shared and historical experiences of discrimination and exclusion, including in shelters and transitional housing. More research is needed on affordable housing models for LGBTQ+ older adults, especially those that exist outside of affordable housing developments. Qualitative interviews with community leaders driving these nontraditional housing models would be particularly helpful for elevating their experiences and insights and building more knowledge about a range of affordable housing models that meet the needs of various LGBTQ+ aging communities, especially those at risk for precarious housing and/or being unhoused.

Increased federal data collection on the housing needs of LGBTQ+ older adults could also help improve knowledge about affordable housing needs and models for LGBTQ+ older adults. The federal government could create uniform standards across agencies engaged in survey data to standardize the inclusion of questions regarding sexual orientation, gender identity, and gender expression. However, such actions should only be taken if strong privacy protections are enacted by Congress to ensure such information cannot be used against individuals.

Much of the literature above also notes the existence (and often persistence) of discrimination across the life course for LGBTQ+ older adults, including in housing. Policies like the Equality Act help enshrine LGBTQ+ protections into existing civil rights laws. Even after the enactment of such protections, more steps are needed to ensure the laws are fully enforced. For example, policy must be enacted to guarantee transgender older adults can access safe and affirming emergency housing. However, that will only be successful if at the same time more resources are given to help transgender-led organizations acquire the necessary funding and capital to create tiny homes, restored homes, or other intentional communities for affordable housing. More funding is also needed to support these nontraditional housing models, which may be especially salient for transgender and BIPOC-led organizations that may have fewer resources (or desire) to create traditional housing developments. Changing zoning laws could also help LGBTQ+ organizations pursue these nontraditional housing models.

Relatedly, many of the affordable housing models (and some other models) limited who constitutes an older adult. Literature discussed housing developments that used 55, 60, or 62 as guideposts for inclusion. Given research on accelerated aging (e.g., Pereira and Banerjee, 2021; Valencia et al., 2023), these ages may exclude communities that experience multiple forms of marginalization across the life course that may elevate or expedite their needs for aging services sooner (e.g., transgender women of color). We reduced the age to 40 for our review, but more attention is needed on how housing can serve communities that experience accelerated aging.

While not specifically a housing model, several articles discussed the importance of training (e.g., Johnston and Meyer, 2017; Putney et al., 2020; Steele, 2022; White and Gendron, 2016), which could reduce the need for LGBTQ+ older adults to relocate. Literature advocated for more trained and culturally responsive providers in non-LGBTQ+ affordable housing spaces (e.g., senior housing, assisted living; e.g., Johnston and Meyer, 2017; Putney et al., 2020; Steele, 2022; White and Gendron, 2016). Moreover, because nondiscrimination laws prohibit designating affordable housing as explicitly or exclusively for LGBTQ+ older adults, some spaces targeting LGBTQ+ older adults may comprise many non-LGBTQ+ older adults—including some who may be less affirming. One article noted that 40% of the residents in one LGBTQ+ affordable housing development were not members of the LGBTQ+ community (Dixon, 2018). Regular culturally responsive trainings and workshops for both staff and residents may mitigate bias toward LGBTQ+ older adults that could help them feel safe to age-in-place in their current housing.

## Limitations

One limitation of this study is that it does not include a comprehensive gray literature scoping review beyond the scholarly databases or community-engaged research. Only gray literature that emerged from the scholarly scoping review was included, in addition to the gray literature and community reports provided by the community partner. Given the paucity of overall literature on this topic, we determined that it was important to incorporate gray literature that emerged from the database searches for this project. Future research could include a more robust and systematic gray literature scoping review outside of scholarly databases to identify additional gray literature, including newspaper articles, community reports, ephemera, and other documents that provide more data about various affordable housing models for LGBTQ+ older adults.

Another limitation is that this project lacks data from community leaders creating nontraditional housing options for LGBTQ+ older adults, especially those who may be unable or unwilling to live in LGBTQ+ affordable housing developments. Future research could include interviews of community members involved in building these spaces, specifically targeting BIPOC and/or transgender older adults and other underrepresented communities, given the dearth of existing research and voices in this space.

## Conclusions

LGBTQ+ older adults experience economic disparities and unique needs for affordable housing given a lifetime of stressors and discrimination, including higher likelihood of poverty and health disparities compared to cisgender and heterosexual older adults (Bouton et al., 2023; Emlet, 2016; Turesky, 2022). Affordable housing developments offer an important option for some LGBTQ+ older adults to secure affordable housing, but they are but one of many potential housing models. And while affordable housing developments dominated the scholarly and gray literature, they cannot remain the primary solution for addressing housing precarity among LGBTQ+ older adults. The need is far greater than the supply, and some evidence suggests that they may not be meeting the needs of a diverse LGBTQ+ aging population. More attention on keeping LGBTQ+ older adults in their homes by providing supports for NORCs or through homesharing could help some LGBTQ+ older adults age-in-place or age-in-community. Homesharing could also provide housing opportunities for LGBTQ+ older adults who need safe, stable, affordable, and supported housing. Cohousing, mobile communities, restored homes, CLTs, ADUs, LEC, and tiny homes also present promising models that merit further research and policy attention. Ultimately, this study underscores the diversity of housing models (and need for more research) that researchers, practitioners, and policymakers could leverage to better understand the multiple avenues for supporting affordable housing for LGBTQ+ older adults.

## Supplementary Material

gnaf103_suppl_Supplementary_Materials

## Data Availability

The protocol for this scoping review was registered on Open Science Framework at https://osf.io/rtcqg.
